# Human Chorionic Gonadotropin Injection to Retrieve Mature Oocytes During Laparoscopic Surgery for Ovarian Tissue Cryopreservation: A Case Report

**DOI:** 10.7759/cureus.82606

**Published:** 2025-04-19

**Authors:** Hiroyuki Okimura, Akika Iwata, Mari Kawamata, Yuko Izumi, Hisashi Kataoka, Fumitake Ito, Taisuke Mori

**Affiliations:** 1 Obstetrics and Gynecology, Kyoto Prefectural University of Medicine, Kyoto, JPN

**Keywords:** fertility preservation, human chorionic gonadotrophin, oncofertility, oocyte cryopreservation, pediatric medulloblastoma

## Abstract

For young women with cancer facing the risk of infertility due to upcoming chemotherapy or radiotherapy, embryo cryopreservation (ECP), oocyte cryopreservation (OCP), and ovarian tissue cryopreservation (OTC) have been proposed. For some adolescent and adult patients who need immediate initiation of cancer treatment as well as prepubertal patients, only OTC is an option for fertility preservation. However, OCP is recommended for patients who can choose both OCP and OTC because the live birth rate after OTC is still insufficient. In this case, we were able to perform OCP as well as OTC with human chorionic gonadotropin (hCG) injection before surgery for a pubertal girl. The parents of a 15-year-old girl undergoing radiotherapy and chemotherapy (vincristine) after brain surgery for medulloblastoma approached our department to request fertility preservation for their daughter. Her menstruation had stopped, and maintenance chemotherapy (cisplatin, vincristine, cyclophosphamide) was scheduled one month after the current treatment ended. We informed them about the utility of each fertility preservation method and recommended OTC for the patient during her break from chemotherapy. The parents desired OCP because the pregnancy rate for OTC and ovarian tissue transplantation (OTT) was not satisfactory. With magnetic resonance imaging (MRI) showing small follicles and the resumption of menstruation in the patient, we decided to conduct follicle aspiration for OCP during surgery for OTC. Thirty-five hours before laparoscopic oophorectomy for OTC, hCG was injected. During laparoscopic surgery, all three small follicles that were visible were manually aspirated with an 18-gauge needle. Two oocytes, one mature and one degenerated, were retrieved. The mature oocyte and unilateral ovarian tissue were cryopreserved. The patient was discharged two days after OTC and returned four days later for maintenance chemotherapy. For young patients with cancer undergoing OTC, hCG administration may be an effective therapeutic option for retrieving mature oocytes during surgery.

## Introduction

Recent advances in cancer treatments and reproductive technologies have heightened interest in the pregnancy prospects of young patients with cancer, creating a field called oncofertility [1]. For young women with cancer facing the risk of infertility due to upcoming chemotherapy or radiotherapy, embryo cryopreservation (ECP), oocyte cryopreservation (OCP), and ovarian tissue cryopreservation (OTC) have been proposed. However, several days of ovarian stimulation, sometimes followed by ovarian hyperstimulation syndrome (OHSS), are necessary for ECP and OCP. Thus, for some adolescent and adult patients who need immediate initiation of cancer treatment as well as prepubertal patients, only OTC is an option for fertility preservation [2,3].

Currently, ovarian tissue transplantation (OTT) is the only method to achieve pregnancy using cryopreserved ovarian tissue. OTT also has the advantage of restoring endocrine function [4]. However, the live birth rate after OTT for fertility preservation is reported to be 21% for pregnancy with in vitro fertilization (IVF) in a recent meta-analysis [5]. For some patients, most of whom are burdened mentally with a cancer diagnosis, this low success rate may be a barrier blocking the decision to undergo OTC. Retrieving mature oocytes from in vitro cultured ovarian tissue is still difficult. In addition, OTT has a risk of retransferring the malignant cells.

Conversely, the live birth rate following ECP and OCP for fertility preservation has been reported to be 41% and 32%, respectively [5]. Although it is difficult to compare these live birth rates between ECP, OCP, and OTC due to differences in patient age, underlying malignant disease, institutes, and country, OCP is recommended for patients who can choose both OCP and OTC, except in the case of a patient preference [2,3].

Human chorionic gonadotropin (hCG) is used to induce ovulation and maturation of oocytes in fertility treatment and IVF cycles. It has been reported that oocytes in the metaphase II (MII) stage could be efficiently retrieved with a lag time of 35 to 38 hours from the ovulation trigger with hCG to ovum pick-up (OPU) [6]. This lag time would not be a significant delay for cancer treatment. Along with the objective of promoting oocyte maturation, hCG injection before surgery may also have the potential to achieve OCP concurrently with OTC. Here, we report a case in which a mature oocyte was retrieved during laparoscopic surgery for OTC following hCG injection.

## Case presentation

The parents of a 15-year-old girl who was undergoing radiotherapy and chemotherapy (vincristine) after brain surgery for medulloblastoma approached our department and requested counseling for fertility preservation for their daughter. Her medical and family histories were unremarkable. Her menstruation had begun at the age of 12 but stopped during her treatment two months before counseling. She had no history of sexual intercourse. She had complained of headaches, nausea, dizziness, and loss of appetite before brain surgery. After tumor resection, she developed cerebellar mutism syndrome (CMS). She appeared to understand our explanation. She could speak a little and indicate her intention with a few words. She also had pneumothorax and hydrocephalus, for which a ventriculoperitoneal shunt was placed. Maintenance chemotherapy with cisplatin, vincristine, and cyclophosphamide at a dose of >7.5 g/m2 was scheduled one month after the current treatment was complete. This dose of cyclophosphamide corresponds with a high risk of permanent amenorrhea [7].

We informed the parents of the patient of the utility of each fertility preservation method and recommended OTC during her break from chemotherapy. At first, the parents desired OCP as it may provide a greater chance of pregnancy compared with OTC in general. However, OCP could be challenging because the patient was undergoing chemotherapy, and her menstruation seemed to have stopped. In addition, she denied transvaginal puncture and daily injection for ovarian stimulation. Partly due to CMS, she tended not to agree with invasive procedures. Thus, it was difficult to explain the safety, effectiveness, and necessity of transvaginal puncture and ovarian stimulation in detail to the patient.

**Figure 1:**
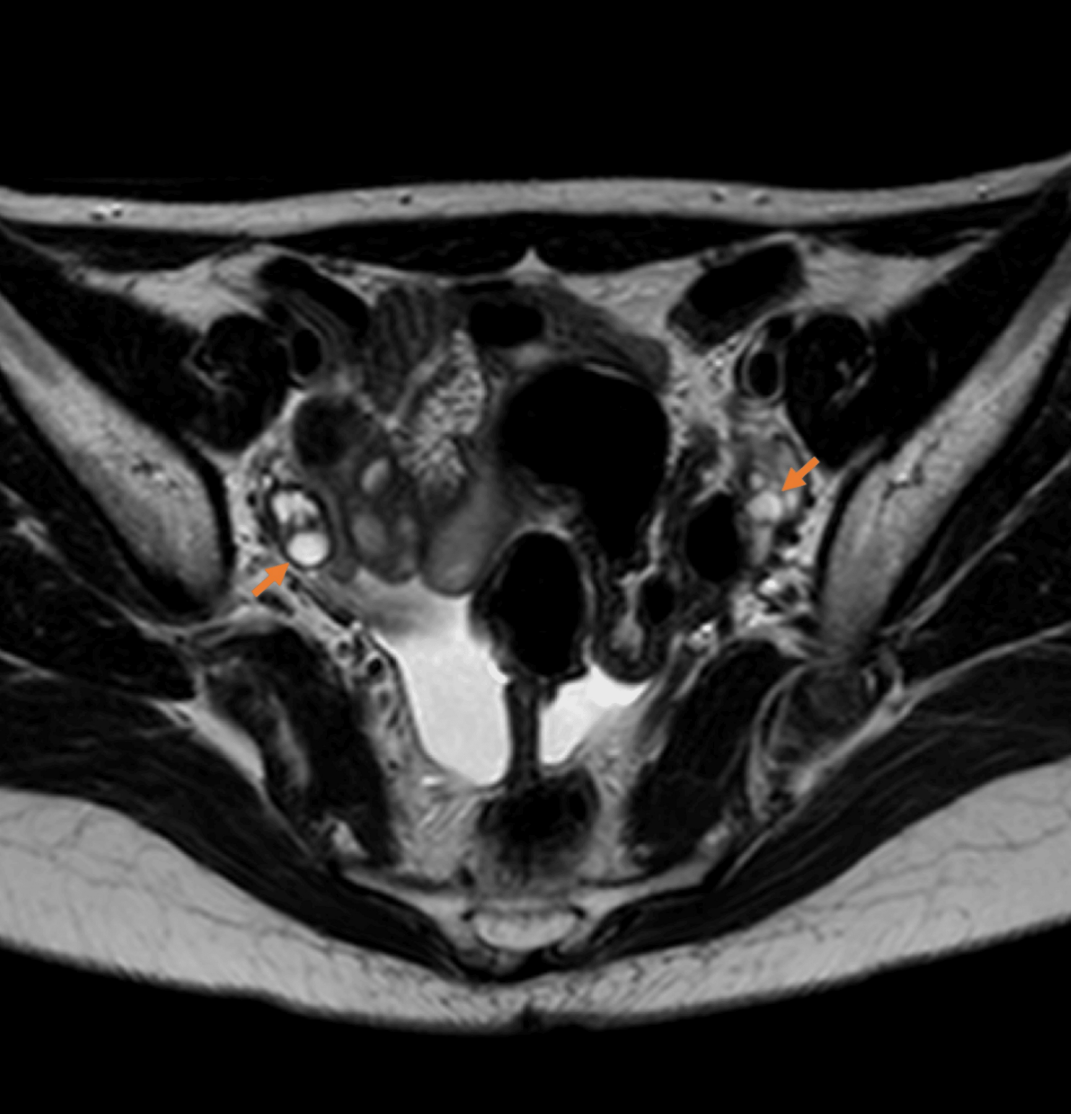
Magnetic resonance imaging (MRI) radiographs showing small follicles (orange arrows)

Transabdominal ultrasonography 21 days after her last administration of chemotherapy showed a right ovarian simple cyst that was 3 cm in diameter. We could detect a few small follicles in both ovaries by employing magnetic resonance imaging (MRI) three days after ultrasonography, although the cyst had disappeared (Figure 1). It was suggested that the cyst may have been a growing follicle that had undergone ovulation before the day of the MRI. Her menstrual cycle resumed 29 days after her last chemotherapy. However, only a few days remained for fertility preservation intervention before the resumption of chemotherapy, and this remaining duration was too short to perform OCP.

We counseled the patient and her parents again and proposed an OPU procedure during surgery for OTC following hCG injection, as they still desired OCP. The patient and her parents showed their assent and consented to the proposal. We discussed the schedule of the day of operation and risks for anesthesia with our surgical department and anesthesiologists to perform OPU at a fixed time when the follicles probably developed into the MII stage after hCG injection. We did not employ ovarian stimulation to avoid multiple follicular growth, which carried the risk of ovarian OHSS that would make it difficult to cut the ovarian cortex into small pieces for cryopreservation.

**Figure 2:**
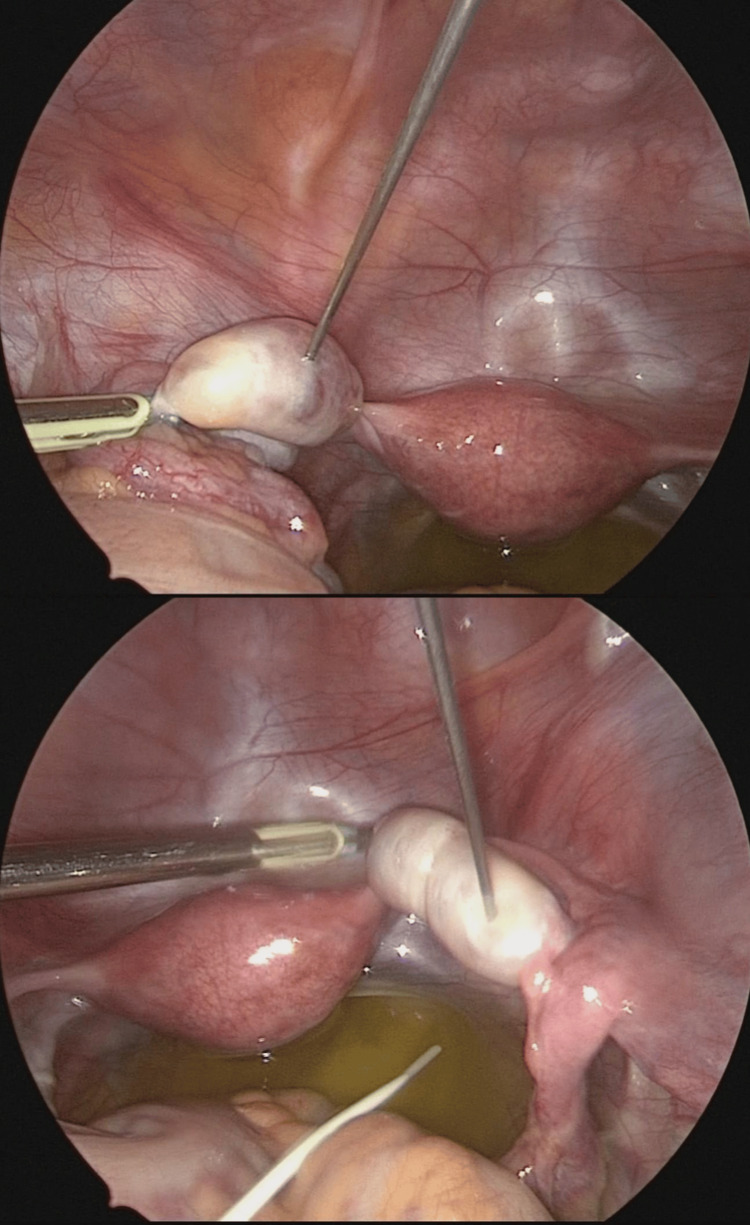
Image showing ovum pick-up during laparoscopic surgery

Thirty-five hours prior to the initiation of laparoscopic oophorectomy, the patient was injected intramuscularly with an hCG dose of 5000 IU. Based on her age, body weight, and number of follicles, we considered this standard hCG dose to be safe and effective. Transabdominal ultrasonography conducted on the day before OTC showed 1.0 cm diameter follicles in the right ovary and 1.1 cm diameter ones in the left ovary. During laparoscopic surgery for unilateral ovarian resection, all three visible follicles were manually aspirated with an 18-gauge needle (Figure 2). Two oocytes, one MII stage and one degenerated, were retrieved. We did not find small follicles in the resected whole ovary or cumulus-oocyte complexes (COCs) in the medium in which the ovarian cortex was cut into small pieces for cryopreservation: COCs are normally observed in ovarian cortices obtained without hCG injection. The mature oocyte and excised right ovarian tissue were cryopreserved by vitrification. The patient was discharged two days after laparoscopic surgery and returned four days later for maintenance chemotherapy.

## Discussion

In this case, we were able to perform OCP as well as OTC with hCG injection before surgery. hCG injection before OTC may have the potential to increase the probability of future pregnancy for young patients with cancer who have no choice but OTC for fertility preservation.

To increase the pregnancy rate for patients undergoing OTC, simultaneous in vitro maturation (IVM) of immature ovarian tissue oocytes (OTO) is being attempted [8]. During ovarian tissue processing for OTC, oocytes may be retrieved from visible follicles and the fluid remaining after ovarian tissue dissection. These oocytes are often immature and need to undergo IVM. IVM has been gaining increasing interest as an alternative to conventional ovarian stimulation to avoid OHSS. For IVM, patients have to be prepared with follicle-stimulating hormone and hCG and/or gonadotropin-releasing hormone in many cases [9]. COCs containing immature oocytes are collected with needle aspiration, followed by in vitro culture until they reach the MII stage. Currently, IVM is considered to be a promising technology with reported maturation rates of up to 84% [10]. On the other hand, Segers et al. have reported the maturation rate of OTO-IVM to be 39 ± 23% [11]. The success rate of OTO-IVM should not be compared with IVM because in OTO-IVM, COCs are collected from small follicles or residual medulla, which have been scraped from the cortex, unlike generic IVM with ovarian stimulation and needle aspiration [12]. In addition, the patient populations are different between the studies investigating OTO-IVM for patients with cancer and IVM for patients with polycystic ovarian syndrome. At the present time, the effectiveness of OTO-IVM is still controversial. In the patient we have described here, we were able to retrieve an MII stage oocyte without IVM.

In IVM, it has been reported that the inclusion of an hCG injection may improve the clinical outcome by dispersing cumulus cells, which may facilitate the identification of COCs and increase the maturation rate [13]. Thus, hCG injection prior to OTC may also have the potential to elevate the success rate of OTO-IVM.

In the current case, the follicle size before hCG injection was small. Usually, the oocyte recovery rate of small follicles is lower compared with medium or large follicles. It is reported that the oocyte and MII stage oocyte recovery rate of small 8-12 mm/0.3-0.9 mL follicles after controlled ovarian stimulation and hCG injection is 63.8% and 37.8% per follicle, respectively [14]. It has also been shown that oocytes derived from small follicles have almost the same capacity for embryo development as those from larger follicles. If only small follicles are visible in the ovaries before OTC, an hCG injection still has the potential to develop mature oocytes capable of becoming embryos.

It has been reported that OTC could be combined with controlled ovarian hyperstimulation (COH) [15]. However, it was reported simultaneously that COH before OTC induced hemorrhagic suffusion and edema in the ovarian tissue, even though there was no effect on the oocyte apoptotic rate. Thus, COH has a risk of making it difficult to process resected ovarian tissue as well as OHSS. Simply adding hCG injection before OTC can increase the possibility of obtaining oocytes that are mature and have better quality for IVM. However, with hCG injection only, we could not retrieve plenty of oocytes. In this case, the number of mature oocytes cryopreserved is only one. To retrieve mature oocytes as well as ovarian tissue in good condition safely from patients undergoing OTC, it should be further elucidated whether combining COH or simply adding hCG with or without OTO-IVM can be applied.

The mature oocyte cryopreserved in this case was retrieved during the patient's break from chemotherapy. Pregnancy should be postponed until the residual chemotherapeutic agent has disappeared from the body of the patient. Although there is no clear guideline on the length of time after chemotherapy that the patient should wait before attempting pregnancy or the OPU procedure, it has been suggested to wait at least one year post-chemotherapy completion before getting pregnant in order to reduce the risk of complications in pregnancy [16]. For this patient, her visit to our department was the last opportunity to consider fertility preservation before chemotherapy resumed. Resumption of chemotherapy in her case would be associated with a high risk of primary ovarian insufficiency. Her menstrual cycle had recovered, and we reasoned that it was feasible to recommend OCP and OTC for her. However, whether the oocyte, as well as ovarian tissue in this case, should be used or not must be discussed based on future evidence.

In the field of oncofertility, patients have various complications related to cancer and cancer treatment. In this case, the patient had developed cerebellar mutism after brain surgery. Postoperative pediatric CMS is transient, and total cerebellar-induced speechlessness or emotional lability occurs after cerebellar or 4th ventricle tumor surgery in children [17]. Due to CMS, it was difficult to confirm how she was thinking about having a child in the future and what she wanted to do for fertility preservation. Every time we talked with her and her parents, we explained what we did repeatedly as she develops into an adult and recovers from CMS. In our facility, patients after OTC are required to come to the hospital at least once a year to confirm continuation of cryopreservation in addition to a regular checkup. Storage will be continued until the patient's wish for OTT or disposition.

## Conclusions

In this case, we were able to perform OCP as well as OTC with hCG injection before surgery without complications such as OHSS. Administration of hCG prior to the OTC procedure may be an effective therapeutic option to retrieve mature oocytes without IVM. Further multicenter trials or registries are needed to elucidate the effectiveness of this method, including determining the live birth rates with retrieved MII stage oocytes, documenting perioperative adverse effects, and long-term outcomes.
